# Functional Outcomes in a Randomized Controlled Trial of Animal-Assisted Therapy on Middle-Aged and Older Adults with Schizophrenia

**DOI:** 10.3390/ijerph19106270

**Published:** 2022-05-21

**Authors:** Chyi-Rong Chen, Chi-Fa Hung, Yi-Wen Lee, Wei-Ting Tseng, Mei-Li Chen, Tzu-Ting Chen

**Affiliations:** 1 Department of Psychiatry, Kaohsiung Chang Gung Memorial Hospital, College of Medicine, Chang Gung University, Kaohsiung 833401, Taiwan; ccr776@cgmh.org.tw (C.-R.C.); chifa@cgmh.org.tw (C.-F.H.); summer31@cgmh.org.tw (W.-T.T.); 2 School of Occupational Therapy, College of Medicine, National Taiwan University, Taipei 100025, Taiwan; 3 Department of Occupational Therapy, Shu-Zen Junior College of Medicine and Management, Kaohsiung 821004, Taiwan; 4 Department of Post-Baccalaureate Medicine, National Sun Yat-Sen University, Kaohsiung 804201, Taiwan; 5 College of Humanities and Social Sciences, National Pintung University of Science and Technology, Pingtung 912301, Taiwan; 6 Department of Nursing, Kaohsiung Chang Gung Memorial Hospital, College of Medicine, Chang Gung University, Kaohsiung 833401, Taiwan; hikare2000@adm.cgmh.org.tw; 7 School of Nursing, National Taipei University of Nursing and Health Sciences, Taipei 112303, Taiwan; meili@ntunhs.edu.tw; 8 Professional Animal-Assisted Therapy Association of Taiwan, Taipei 112303, Taiwan

**Keywords:** animal-assisted therapy (AAT), aging, schizophrenia, physical function, social function

## Abstract

Deficits in cognition, physical, and social functions in adults with schizophrenia may become salient with aging. While animal-assisted therapy (AAT) can benefit physical function in older adults and improve symptoms of psychotic disorders, the effect of AAT on middle-aged patients with schizophrenia is unclear. The current randomized controlled trial aimed to explore the efficacy of AAT for middle-aged patients with schizophrenia. Forty participants were randomly assigned to either the AAT or control group. The AAT group participated in one-hour sessions with dog-assisted group activities once a week for 12 weeks. The controls participated in dose-matched, non-animal-related recreational activities. Both groups remained on their usual psychotropic medication during the trial. Evaluations included the Chair Stand Test (CST), Timed Up-and-Go (TUG) test, Montreal Cognitive Assessment (MoCA), 5-Meter walk test (5MWT), and Assessment of Communication and Interaction Skills (ACIS). The increases in CST repetitions and ACIS scores were larger in the AAT group than in the controls. The two groups did not differ significantly in MoCA scores, TUG performance, or the 5MWT. The AAT group showed a greater increase in lower extremity strength and social skills, but no improvement in cognitive function, agility, or mobility. Further research with more sensitive evaluations and longer follow-up is needed.

## 1. Introduction

Schizophrenia is a severe mental illness that can manifest as positive symptoms, negative symptoms, and cognitive impairment [1]. About 1% of the population is affected by schizophrenia, a quarter of whom are middle-aged and older [2]. Evidence has shown that patients with schizophrenia have increased oxidative stress and accelerated aging [3]. Their life expectancy is approximately 14.5 years shorter than that of the general population [4]. Research has also shown poorer physical health and sedentary lifestyles among patients with schizophrenia [5,6]. Recent studies have noted that muscular strength may be a predictor of psychotic progression of schizophrenia [7]. Therefore, there is an urgent need to develop efficient treatment to improve the physical health of patients with schizophrenia.

Cognitive impairment is one of the core deficits of schizophrenia, and plays an important role in functional recovery [8]. Recent evidence has shown that middle-aged patients with schizophrenia display accelerated loss of white matter [9], and may have increased risks of developing dementia [10]. They also experience significant physical and psychosocial challenges, and are at a higher risk of admission to nursing homes and early institutionalization [11,12]. Antipsychotic medication is effective at alleviating positive symptoms [1]; however, it is generally less effective for improving cognition [13].

Furthermore, deficits in communication and interpersonal skills have also been widely documented in patients with schizophrenia [14,15], and social skills predicted their vocational functions [16]. A previous study noted a negative association between social skills and age in patients with schizophrenia spectrum disorder, that is, their social skill deficits persisted from the onset of illness into older lives [17]. Additional variables, such as long-term hospitalization and cognitive impairment, may adversely contribute to decreased social performance in this population [17,18]. Hence, it is important to explore non-pharmacological ways to improve cognitive and interpersonal functions among middle-aged and older adults with schizophrenia.

Animal-assisted therapy (AAT) has previously been applied to patients with dementia and schizophrenia [19,20]. Animal-assisted therapy generally includes a therapist, an animal (typically a dog), and an animal handler (breeder of animal) to facilitate enhanced interactions between patients, animals, and personnel through planned, structured, and goal-oriented therapeutic activities [19,21]. It is hypothesized that animals can serve as emotional mediators to provide a sense of support and companionship [22,23]. The results of a meta-analytic review revealed that dog ownership is associated with reduced mortality, especially in patients with prior stoke. The authors claimed that the effects were possibly driven by the increased physical activity associated with dog ownership [24]. In a recent systematic review, Rodríguez-Martínez et al. (2021) found that AAT has been applied to several neurological diseases (e.g., dementia, stroke and spinal cord injury), resulting in improvements in motor and physical ability, as well as mental and behavioral health [25]. However, few studies have addressed the efficacy of AAT in patients with schizophrenia.

Previous non-randomized clinical trials that recruited middle-aged and older individuals with severe mental illness found that AAT might improve depression, activities of daily living, cognition and quality of life [26,27]. One randomized controlled trial noted a promising effect of AAT for older adults with schizophrenia on social and adaptive functions [18]. In order to increase the quality of care, Chen et al. (2021) developed a structured and diverse AAT treatment program with an interdisciplinary approach. The trial recruited middle-aged and older adults with schizophrenia and found that AAT improved symptomology and emotion [28]. Clinical trials of AAT have found improvements in positive symptoms [29], emotional status [23], and adherence to treatment [23] in adult patients with schizophrenia. However, other studies found no significant effect on quality of life [23,30] and general psychopathology symptoms [30]. A recent systematic review that included seven randomized controlled trials found that AAT studies on patients with schizophrenia were relatively small-scale trials and may be prone to methodological limitations [20]. As a consequence, the current evidence regarding the efficacy of AAT for patients with schizophrenia is scarce and inconclusive. As such, the aim of the present study was to explore the effect of AAT on cognitive, physical, and social functions among middle-aged and older patients with schizophrenia.

## 2. Materials and Methods

### 2.1. Design

This study was a parallel-group, randomized controlled trial. The participants were recruited from a day-care center and rehabilitation wards. Stratification was performed to control for possible confounding factors due the differences in functional capability between participants. After obtaining informed consent, 40 middle-aged and older participants with schizophrenia were randomly assigned to the intervention group (AAT group) or the control group. The allocation ratio was 1:1. The randomization sequences were performed with an online randomizer (www.randomiser.com, accessed on 28 July 2020) by an external clinic. The random numbers indicating group assignment were kept in opaque, sealed envelopes, which were only opened after the baseline assessment. To ensure safety and quality of intervention, participants in the AAT group were further divided into two smaller groups (groups A and B) based on ward. Both groups received the same treatment.

### 2.2. Participants

This study was conducted in the psychiatric rehabilitation ward and the day-care center of a medical center in Taiwan. All eligible participants who met the following criteria were recruited: (1) diagnosis of schizophrenia according to the fifth edition of the Diagnostic and Statistical Manual of Mental Disorders, (2) age over 40 years, and (3) stable health condition based on clinician evaluation. The exclusion criteria were as follows: (1) severe cognitive impairment, such as aphasia, or inability to follow three-step instructions, (2) allergies to animals, (3) history of asthma, (4) confirmed diagnosis of coagulation disorders, (5) presence of symptoms of dog-related phobia, anxiety, or obsessive–compulsive disorder, and (6) participation in other clinical trials in the past 6 months.

### 2.3. Measures

#### 2.3.1. Montreal Cognitive Assessment (MoCA)

The MoCA is a 30-point scale that evaluates visuospatial ability, executive functions, naming, attention, language repeat, language fluency, delayed recall memory, abstraction, calculation, and orientation. The total score indicates global cognitive function. Evidence has shown that the MoCA is sensitive to detect cognitive impairment in patients with schizophrenia [31]. The Taiwanese version of the MoCA has been previously validated [32]. In the current study, a trained and licensed evaluator (TWCHERO42959-01) performed all of the MoCA tests.

#### 2.3.2. Chair Stand Test (CST)

The CST is a performance-based test, which requires participants to rise from a straight sitting position to a full standing position with their arms folded across their chest as many times as possible within a 30 s period. The test was completed on a seat placed near a wall. The number of repetitions was recorded. Higher scores represented better lower body strength [32].

#### 2.3.3. Timed Up-and-Go (TUG)

The TUG is a widely used performance-based test that requires participants to stand up, walk, turn, and sit down. The time to perform the test is recorded in seconds. A lower score indicates better agility [33]. This study applied the 8-feet TUG test described by Jones and Rikli [34]. The TUG has previously been used as an outcome measure to examine the effect of an exercise program in patients with schizophrenia [35] and has good reliability (Cronbach’s α = 0.95).

#### 2.3.4. 5-Meter Walk Test (5MWT)

The 5MWT is conducted to assess mobility. This test assesses the participant’s walking speed in meters per second. To control for the acceleration and deceleration of walking, a 7 m walking distance was required for participants to complete the test. However, only the time to complete the 5 m in the middle of path was recorded [36].

#### 2.3.5. Assessment of Communication and Interaction Skills (ACIS)

The ACIS was used to assess communication and interpersonal skills. The ACIS is an observational assessment that consists of three domains, that is, body, information exchange and relationship. A higher score indicated better communication and interpersonal skills [37]. The Chinese version of the ACIS was adopted with acceptable test–retest reliability and good internal consistency [38].

### 2.4. Interventions

Both the AAT group and the control group continued their usual care programs, including pharmacotherapy, occupational therapy, recreational activities, and psychosocial therapies. During the 12-week intervention period, the participants in the AAT group attended additional one-hour AAT sessions. The controls participated in non-animal-related group activities for the same period. Each AAT session was led by a registered animal-assisted therapist, in cooperation with an occupational therapist specialized in mental illness rehabilitation, a therapy dog, and the breeder of the dog. The therapy dogs and their breeders were all trained by the Professional Animal-Assisted Therapy Association of Taiwan. The dogs were Corgi (5 years old), Labrador Retriever (11 years old), Maltese (8 years old), and Shiba Inu (8 years old).

The AAT therapy was conducted in a wide and quiet room. Each session involved a 15 min warm-up session, such as greetings, introduction of the therapy dogs by their breeders, and session orientation. Subsequently, the 45 min main therapeutic activities were carried out. These therapeutic activities were categorized into the following four types: physical activities, cognitive activities, social activities, and activities for positive emotion. The rationale and goal of each type of activity are published elsewhere [28]. Brief descriptions of the activities are listed in Table 1. Followed by the therapeutic activities, a 5 min feedback session was provided by the therapist. Participants were also encouraged to express their feelings about the therapy session with the dog.

### 2.5. Statistical Analysis

The Shapiro–Wilk test was applied to test the normality of the data. As the data were non-normally distributed, a non-parametric statistical procedure was used. Continuous variables were described as the median and interquartile range (IQR), and were compared using the Mann–Whitney U test.

## 3. Results

### 3.1. Background Characteristics

The background characteristics of the participants have been previously published [28]. There were 40 participants, 55% of whom were female. The median age was 54.6 years. At baseline, there were no significant differences between the AAT group and the control group in MoCA scores (*p* = 0.350), CST (*p* = 0.663), TUG (*p* = 0.579), or 5MWT (*p* = 0.449) (Table 2).

### 3.2. Pre–Post Improvements

Regarding physical performance outcomes, there was a significant difference in the CST performance of the AAT group pre- and post-intervention compared to that of the control group (*p* = 0.007). The AAT group showed more improvement in repetitions of the CST in the testing period. The change in the ACIS score of the AAT group was significant compared to that of the control group (*p* < 0.001). Post-intervention, the ACIS total scores of the AAT group were more significant than those of the controls (*p* = 0.003). However, there were no significant differences in the MoCA scores (*p* = 0.774) and TUG (*p* = 0.598) and 5MWT performances (*p* = 0.168) before and after the intervention. There were no significant differences between the groups in the test results of cognitive and physical functions posttest (Table 2).

## 4. Discussion

This study examined the cognitive, physical, and social functions associated with AAT, an emerging augmented therapy regimen, in middle-aged and older adults with schizophrenia. In addition to the benefits on psychopathology and emotion demonstrated previously [28], the current report found that the AAT also improved lower extremity muscular strength, communication and interpersonal skills. However, there was no significant improvement in global cognition, agility, or walking speed.

Our study revealed no significant difference in global cognitive function between the two groups. Our findings were similar to those of a previous meta-analysis regarding AAT on patients with dementia [19,39,40]. However, a clinical trial exploring the effects of combining AAT with play therapy revealed a slight improvement in the Mini-Mental State Examination total score of older adults after eight weekly sessions [41]. However, this study had the methodological limitations of all pretest–posttest control group studies. The effect of AAT on cognitive functions, such as remembering the names and hobbies of dogs, recalling how to interact with dogs, and practicing the process of taking care of dogs, might be insufficient to generate cognitive improvement in adults with salient cognitive impairment. However, our promising results suggest that the effect of AAT on cognitive functions warrants further investigation.

To the best of our knowledge, this study was the first randomized controlled trial to evaluate the effect of AAT on physical functions among middle-aged and older patients with schizophrenia. The results revealed significant improvements in lower extremity muscular strength after AAT, compared to just recreational activities. This may be underpinned by the following mechanisms. First, interacting and playing with therapy dogs might improve physical activity in older adults [24], thus leading to improvement in muscular strength. Second, the therapeutic program in the current trial included physical activities such as walking and playing with the dogs. The performed activities required gross motor movements, such as squatting down to groom and dress the dogs, which might also contribute to improving lower extremity muscular strength. Third, interactions with animals are known to release oxytocin in people [42,43], thereby reducing negative symptoms, such as a lack of motivation to perform physical activities [44], thus leading to an improvement in physical activity participation and physical functions. However, these possible mechanisms still require further investigation.

Our study revealed no significant differences in agility and mobility between the two groups. In a pilot randomized controlled trial, Grubbs et al. (2016) enrolled older adults and combined AAT with aerobic exercise and resistance training. Compared to aerobic exercise and resistance training alone, exercise augmented with AAT improved hand grip strength [45]. In contrast to our findings, the work by Grubbs et al. (2016) revealed improvements in agility and walking speed following AAT; however, no significant difference was found in lower extremity muscular strength [45]. The difference in the treatment program might account for this discrepancy. In the study by Grubbs et al. (2016), exercise was conducted throughout the program, and the time and frequency of exercise were more than those in our AAT program. However, the optimal involvement of the animal and frequency of the intervention remain unclear and warrant further investigation.

The current study demonstrated significant improvements in communication and interpersonal skills of the AAT group, compared to the control group. A previous study found that their AAT program revealed similar results [18]. The enhancement of interpersonal relationships in participants may be explained by the following mechanisms. First, the dog created a novel “object” for transference; thus, the participants might be reminded of memories of happiness, share feelings and interact with the dog [18]. Second, the dog performed the role of a social catalyst, and provided support and accompanied the participants [22,23], leading to more interactions with the dog and participants. Last, a previous study supported the efficacy of AAT on negative symptoms [28]; as a possible mediator, the increase in motivation toward social interaction, improvement in facial affect and less social withdrawal might have further contributed to the better quality of social interactions in patients with schizophrenia.

This study has many strengths. First, this study incorporated a multidisciplinary approach including an animal-assisted therapist and occupational therapist; the combination of their unique knowledge and skills might help in determining the most beneficial treatment program. Second, there was no drop out in the current study, which implies the feasibility of the AAT program for middle-aged and older adults with schizophrenia. However, there are also several limitations, which should be discussed. First, due to the nature of the AAT intervention, it was impossible to blind the participants and therapists to the group. Moreover, this study was a small-scale trial without follow-up assessment; thus, the results should be interpreted with caution. Further research with a larger sample size and longer follow-up is recommended. In addition, studies including more domain-specific cognitive assessments (e.g., working memory and social cognition) and more comprehensive physical fitness tests (e.g., upper and lower extremity strength, and cardiorespiratory fitness) are warranted.

## 5. Conclusions

Animal-assisted therapy can improve lower extremity strength and social functions in middle-aged and older adults with schizophrenia. Animal-assisted therapy might be a beneficial augmented therapy regimen; however, further research is still needed in order to better understand the characteristics of the effective elements of AAT.

## Figures and Tables

**Table 1:** Contents of animal-assisted therapy.

Type of Activity	Contents of Activities
Physical activity	Walking the dog
	Handling, feeding, grooming, and dressing the dog
	Doing exercises with the dog (warm-up exercise, brisk walking, and stretching exercise)
Cognitive activity	Questions and answers (names or hobbies of dogs)
	Training the dog, such as gaining the focus and attention of the dog, training the dog to come back when called, teaching the dog to sit, stay, and lie down
	Orienting the content of activity
	Playing cognitive games, such as puzzles, triangles, and memory cards
	Writing the worksheets
Social activity	Introducing, greeting, praising, thanking, helping and talking to each participant
	Introducing, greeting, praising, thanking, helping and talking to the dog
	Making appropriate physical and eye contact
	Cooperating in the games and tasks with the dog
Activity for positive emotion	Touching the dog
	Singing songs
	Massaging the dog
	Head, back, and body massaging for each participant
	Playing with the dog, such as finding the treats, and playing ball, loop, and games
	Artistic creation (dot art and coloring pages)

**Table 2:** Median and interquartile range of outcomes and changes in scores pretest and posttest.

		AAT Group (*n* = 20)	Control Group (*n* = 20)	
		Median (IQR)	Median (IQR)	*p* Value
MoCA (score)	Pre	22.50 (11.25)	18.50 (7.50)	0.350
	Post	23.50 (8.00)	20.50 (6.75)	0.472
	Change	1.00 (4.50)	1.00 (3.00)	0.774
CST (repetition)	Pre	11.50 (3.00)	11.50 (6.75)	0.663
	Post	13.00 (8.25)	12.50 (5.75)	0.233
	Change	0.50 (5.75)	−1.00 (2.75)	0.007 *
TUG (second)	Pre	8.81 (2.54)	8.27 (2.11)	0.579
	Post	7.72 (2.72)	7.75 (2.57)	0.839
	Change	−0.22 (1.45)	−0.52 (2.29)	0.598
5MWT (m/s)	Pre	1.13 (0.44)	1.30 (0.90)	0.449
	Post	1.24 (0.76)	1.13 (0.88)	0.570
	Change	0.11 (0.50)	−0.12 (0.90)	0.168
ACIS (score)	Pre	65.00 (10.50)	62.00 (10.50)	0.461
	Post	71.50 (6.00)	65.00 (12.50)	0.003 *
	Change	5.00 (7.70)	0.50 (2.60)	<0.001 **

* *p* < 0.01; ** *p* < 0.001. AAT, animal-assisted therapy; MoCA, Montreal Cognitive Assessment; CST, Chair Stand Test; TUG, Timed Up-and-Go; 5MWT, 5 min walk test; ACIS, Assessment of Communication and Interaction Skills.

## Data Availability

Not applicable.
